# R3D-2-MSA: the RNA 3D structure-to-multiple sequence alignment server

**DOI:** 10.1093/nar/gkv543

**Published:** 2015-06-05

**Authors:** Jamie J. Cannone, Blake A. Sweeney, Anton I. Petrov, Robin R. Gutell, Craig L. Zirbel, Neocles Leontis

**Affiliations:** 1 Institute for Cellular and Molecular Biology, and the Center for Computational Biology and Bioinformatics, The University of Texas at Austin, Austin, TX 78712, USA; 2 Department of Biological Sciences, Bowling Green State University, Bowling Green, OH 43403, USA; 3 European Molecular Biology Laboratory, European Bioinformatics Institute, Wellcome Trust Genome Campus, Hinxton, Cambridge, CB10 1SD, UK; 4 Department of Mathematics and Statistics, Bowling Green State University, Bowling Green, OH 43403, USA; 5 Department of Chemistry, Bowling Green State University, Bowling Green, OH 43403, USA

## Abstract

The RNA 3D Structure-to-Multiple Sequence Alignment Server (R3D-2-MSA) is a new web service that seamlessly links RNA three-dimensional (3D) structures to high-quality RNA multiple sequence alignments (MSAs) from diverse biological sources. In this first release, R3D-2-MSA provides manual and programmatic access to curated, representative ribosomal RNA sequence alignments from bacterial, archaeal, eukaryal and organellar ribosomes, using nucleotide numbers from representative atomic-resolution 3D structures. A web-based front end is available for manual entry and an Application Program Interface for programmatic access. Users can specify up to five ranges of nucleotides and 50 nucleotide positions per range. The R3D-2-MSA server maps these ranges to the appropriate columns of the corresponding MSA and returns the contents of the columns, either for display in a web browser or in JSON format for subsequent programmatic use. The browser output page provides a 3D interactive display of the query, a full list of sequence variants with taxonomic information and a statistical summary of distinct sequence variants found. The output can be filtered and sorted in the browser. Previous user queries can be viewed at any time by resubmitting the output URL, which encodes the search and re-generates the results. The service is freely available with no login requirement at http://rna.bgsu.edu/r3d-2-msa.

## INTRODUCTION

Structured RNA molecules, like most proteins, owe their functions to the unique three-dimensional (3D) structure(s) that they form. Changes in RNA or protein sequences can alter 3D structure, folding, dynamics or interactions with other molecules, and consequently modify functional properties. The first step in understanding how RNA sequence variation affects function is to understand which changes in sequence largely preserve structure, and are thus likely to be neutral, as evidenced by sequence variation across different organisms. The discovery of the nucleic acid double helical structure implied the geometric similarity, or ‘isostericity’, of the cis Watson–Crick (WC) basepairs (AU, UA, GC and CG) and suggested that compensating nucleotide changes at positions forming WC pairs preserve the helical structure and thus are generally neutral with regard to function. These ideas concerning RNA sequence variation of helical regions were applied in the 1960s to compare and align a handful of sufficiently divergent transfer RNA (tRNA) sequences to deduce a common set of WC basepairs shared by all members of the tRNA family, i.e. the well-known ‘cloverleaf’ secondary (2°) structure. The comparative sequence approach was subsequently extended to the 5S, 16S and 23S ribosomal RNAs (rRNAs) to accurately determine their WC pairings (2° structure) and some of the long-range pairs forming ‘pseudo-knots’, long before their 3D structures became available (1).

In the last 20 years, there has been a revolution in RNA 3D structure determination, with the number of RNA structures growing from ∼30 in 1995 to over 800 unique structures today, including complete ribosome structures representing almost all the major phylogenetic domains (2). These structures confirmed the WC basepairs deduced by comparative sequence analysis (1), but revealed many more interactions, including non-WC basepairs of all types (3,4), surprising cross-strand and long-range base-stacking interactions, and local and long-range base-backbone interactions that further stabilize folded RNA structures (5,6). Non-WC basepairs comprise more than one-third of all basepairs in structured RNAs (7) and define 3D motifs found in nominally single-stranded regions (8). The 3D structures revealed that most hairpin, internal and junction loops, as well as the ‘single-stranded’ regions capping helices and linking domains, are in fact highly structured (9). Many of these form recurrent motifs that perform similar functions in different contexts (8,10,11). Thus, the concept of many-to-one mapping of sequences to structures first elucidated for WC-paired regions of RNAs appears to also apply to 3D motifs structured by other interactions. Although it is probably not possible to solve enough 3D structures to identify all the sequences that can form each type of RNA 3D motif, that may not be necessary, because we can map instances of 3D motifs from structures to sequence alignments by establishing nucleotide-level mappings between well-resolved 3D structures and high-quality sequence alignments. In this way, the value of these two complementary types of data can be enhanced for addressing questions regarding RNA structure, function and sequence evolution.

There are, however, technical barriers to establishing these mappings in a seamless and easy-to-use way, and thus it is not surprising that these data have remained in their respective silos. RNA 3D structures are archived in the PDB and are served up by NDB with annotations of nucleotide-level interactions (2), while curated RNA sequence alignments are scattered across a large number of boutique databases, although efforts are underway to unify these resources (12). Rfam has played an important role by providing alignments for new RNA families as they are revealed in genomes (13). Rfam automatically extracts new RNA sequences from genomes and clusters and aligns likely homologs into families. Rfam lists any 3D structures from PDB for RNA families that contain an instance of that family, but these listings only include the relevant chain(s) within the 3D structure file(s) and the starting and ending nucleotides. They do not provide nucleotide-level mappings that are needed to match the columns of an alignment with corresponding nucleotides of the 3D structure(s).

The RNA 3D Structure-to-Multiple Sequence Alignment (R3D-2-MSA) server aims to bridge this gap, starting with the rRNAs, which represent the largest structures available, and thereby to facilitate efforts to reveal structural, dynamical and functional constraints on sequence that can lead to new generalizations and principles of RNA folding and evolution. The R3D-2-MSA server allows one to query RNA sequence alignments by direct reference to nucleotide positions in RNA 3D structures, thus facilitating the retrieval of likely sequence variants of known RNA 3D motifs. Its scope currently includes the highest-quality 3D structures of rRNAs from bacterial, archaeal and eukaryal, plus mitochondrial and chloroplast ribosomes, and associated alignments from the Comparative RNA Web (CRW) Site (14). In addition to column-oriented extracts of alignment data, links are provided to 2° structures and to the complete alignments.

## MATERIALS AND METHODS

### Implementation

**Figure 1.**
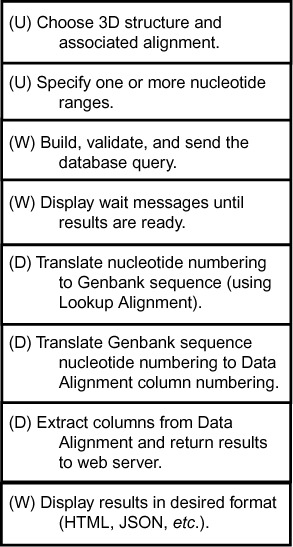
Block diagram of the R3D-2-MSA process. The key high-level elements of the process are described in order from top to bottom. The components that act during each element are noted: U: user; W: web server; D: database server.

The R3D-2-MSA server has two main components, a web-based front end and a database back end. Users build and submit queries and retrieve data through the front end, which handles manual or programmatic input. R3D-2-MSA provides an Application Program Interface (API) for programmatic input. The back end transforms the input queries into the desired result sets and returns that data to the user. The data flow is summarized in Figure 1, where ‘U’ refers to the user, ‘W’ to the web-based front end and ‘D’ to the database back end.

#### Web-based front end

The user-facing component is a python Flask server, hosted at BGSU, which creates the input and results pages. All web pages are styled using the CSS Bootstrap framework and use JavaScript to provide validation of inputs for rapid feedback. The python server parses the requested nucleotide ranges and performs additional validations. After data validation, a stored procedure (described below) is called on the database server to extract and summarize the alignment, whereupon the python server produces the result page, in response to manual input, or returns a JSON object, upon programmatic input. The results page uses the DataTables JavaScript library for efficient filtering and display of the results and the PV JavaScript viewer to interactively display regions of the 3D structure.

#### Database back end

The back end component is located at the University of Texas at Austin and is an implementation of the RNA Comparative Analysis Database or ‘rCAD’ (15), using SQL Server 2008 R2. The rCAD database encodes the sequence alignment data in a format that promotes effective storage and efficient updates (15). The R3D-2-MSA server uses three particular functional features of SQL Server to enhance its performance. The first functionality is the parameterized stored procedure, which combines with our data type and value validations to ensure that all submitted queries comprise valid input data. Encapsulating the queries inside stored procedures minimizes the communication pathways between the web and database server components. One stored procedure call populates the input page with information about 3D structures, alignments and chains, while a second processes the user queries and returns the requested set of sequence fragments and associated metadata, including references to the NCBI Taxonomy (16). As all calculations are done ‘on-the-fly’, R3D-2-MSA has no ‘batch’ mode and therefore is not available as a standalone program. The limit of 50 nucleotides per range in queries is due to a recursion limit on the number of alignment columns returned within the SQL Server. The object-level security features of SQL Server implement the ‘principle of least privilege’, so that the requests made by the web server interact only with a small set of database objects and in a limited manner. Finally, the filtered index option is applied to improve the performance of all alignment-based queries. While all of the alignment data in rCAD is stored in a single table, each of the alignment datasets that R3D-2-MSA accesses has its own index, containing only the data for that alignment, which rCAD will preferentially use to more quickly return the corresponding data. The alignment datasets are described in the next section.

### Sequence alignments

**Figure 2.**
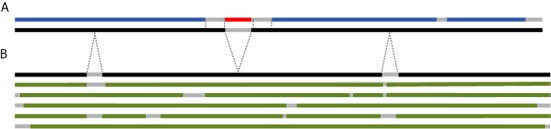
Schematic representation of Lookup and Data Alignments used by R3D-2-MSA. (**A**) Lookup Alignment. Sequences from 3D structures (blue) are aligned to the most similar Genbank sequence, the ‘lookup sequence’, shown in black. An inserted sequence (red) is found in this 3D sequence which has no corresponding region in the lookup sequence, shown by the gap in the (black) lookup sequence. The inserted sequence replaces parts of the biological sequence, shown as gaps in the (blue) PDB sequence. (**B**) Data Alignment. The lookup sequence (black) and five data sequences (green) are shown. The lookup sequence is in both the Lookup and Data alignments and provides the mapping from the PDB sequence positions to the columns of the alignments. Gray dotted lines indicate the positions of gaps and insertions in the lookup sequence in the two alignments.

The R3D-2-MSA server uses a two-step process to map the nucleotides of RNA molecules in PDB files to the columns of the multiple sequence alignments (MSAs), or ‘Data Alignments’, that contain all the sequences of each type of RNA molecule that are queried to provide sequence variants in response to user queries. This process requires a second set of alignments that we call the ‘Lookup Alignments’, to distinguish them from the ‘Data Alignments’. There is a distinct Lookup Alignment for each Data Alignment, for example, distinct ones for bacterial SSU and for eukaryal SSU rRNAs. The Lookup Alignments contain the RNA sequences from the 3D structures selected for use by the R3D-2-MSA server, manually aligned to the most similar RNA sequence found in GenBank. The same GenBank sequences are also in the Data Alignments. The relationships between sequences in the Lookup and Data Alignments are shown in Figure 2. Both the Lookup and Data Alignments are stored within rCAD, keyed and indexed to support data retrieval by the R3D-2-MSA server.

#### Lookup alignments

The R3D-2-MSA server uses a series of ‘Lookup Alignments’ to translate from the PDB entry sequences to the Genbank-sourced sequences found in the ‘Data Alignments’ (described below). Each PDB sequence is matched with the most similar sequence found in Genbank, and the pair is added to the Lookup Alignments; for many structure-sequence pairs, an exact match is available. When there is no identical match (e.g. for engineered sequences), preference is given to the most similar sequence having a minimum number of insertion–deletion events. Usually a PDB sequence can be aligned with high accuracy to its Genbank counterpart; this is done manually, using the 3D structure to resolve ambiguities in regions where the sequence similarity is below 100%. The 22 PDB structure chains included in the initial release of R3D-2-MSA contain 30 893 PDB nucleotides, of which a total of just 10 nucleotides (0.03%) could not be mapped onto their respective Genbank equivalent sequences. These 10 nucleotides are unavailable for use as query endpoints. Figure 2A shows a pair of sequences in a Lookup Alignment, with the PDB sequence represented schematically in blue, its Genbank match in black and insertion–deletion events shown as gray gaps. The PDB sequence contains an engineered addition, shown in red, that is not present in the Genbank reference sequence. The addition, which could, for example, be a protein-binding hairpin added to aid crystallization, partially replaces nucleotides found in the Genbank sequence, which are represented by gaps surrounding the red insert. The R3D-2-MSA server is unable to use nucleotides in red or missing regions of the PDB sequence as range endpoints. Users can still query this region of the alignment by selecting endpoints that bridge the insert. Also, the Genbank sequence is longer at the 3’ end, which has no effect on this server. The net effects of the Lookup Alignments are (i) to map the input position numbers into the ‘natural’ numbering system (i.e. consecutive positive integers) of the lookup sequence, which is used to select the columns for each range in the query, and (ii) to keep engineered sequences sequestered from alignments of biological sequences, so that they do not affect any analytical work.

#### Data alignments

The representative sequence alignments used by the R3D-2-MSA server are generated semi-automatically and manually curated to meet the following criteria: (i) reliable full-length sequences, (ii) provenance from genomes, whenever possible, (iii) high taxonomic diversity and (iv) minimal redundancy. Structure-based alignments represent the optimal juxtapositioning of nucleotides that are likely to occupy equivalent positions in the 3D structures, and are superior to alignments based on sequence identity alone (14). Given alignments that have matured as the comparative 2° structure models were refined, new sequences are aligned using the programs CRWAlign-1 and CRWAlign-2 (17,18), which reliably align sequences that are similar to previously aligned ones; moreover, the programs identify regions that lack sufficient similarity for automated alignment and flag them for manual curation. The quality of the alignments is continually evaluated by ensuring that newly aligned sequences satisfy the maximum number of sequence and structural constraints.

Figure 2B provides a schematic representation of the Data Alignment corresponding to the Lookup Alignment in Figure 2A. The lookup sequence is present in both the Lookup and Data Alignments and is again shown in black in Figure 2B. The dotted gray lines map the corresponding regions across the gaps in the Lookup and Data Alignments. The other sequences of the Data Alignment are shown in green. Each of the sequences has unique combinations of insertion–deletion events (shown as gray-colored gaps in the schematic lines).

#### Alignment usage

The use of two alignment classes, the ‘Lookup’ and ‘Data’ alignments, enables the R3D-2-MSA system's queries. User queries provide position numbers using the PDB numbering system. These are translated to the consecutive integer numbers of the Genbank lookup sequence and are used as input by the database code, which collects the requested sequence fragments from the data alignment. The translation process is transparent to the user.

### 3D structures

The sequence alignments are accessed by reference to the corresponding nucleotides from representative high-quality, atomic-resolution 3D structures. These structures are identified by the data pipeline operating at BGSU to construct the non-redundant (NR) set of structures (http://rna.bgsu.edu/rna3dhub/nrlist), based on a set of previously described structural criteria (19) and made available through NDB (2). Currently, we support 16 structures and 12 alignments, as shown in Table 1. The structures span rRNA from the small and large ribosomal subunits and all three major phylogenetic domains, as well as the chloroplast and mitochondrial organelles. For simplicity, we refer to the rRNA of the Small ribosomal Sub-Unit as ‘SSU rRNA’, regardless of its size, which can vary from 12S for SSU from animal mitochondria to 18S for those of eukaryotes. Similarly, ‘LSU rRNA’ refers to the long rRNA of the Large ribosomal Sub-Unit, which can range in size from 16S to 28S. The SSU and LSU rRNAs are stored in separate sequence alignments and can only be queried separately. Thus, it is not possible at present to simultaneously query nucleotides belonging to the SSU and others belonging to the LSU. While the 5S rRNA is also found in the LSU, it is a distinct molecule from the long LSU rRNA, and therefore the sequences of the 5S rRNA are stored in a separate Data Alignment and must also be queried separately from the other rRNAs.

**Table 1.**
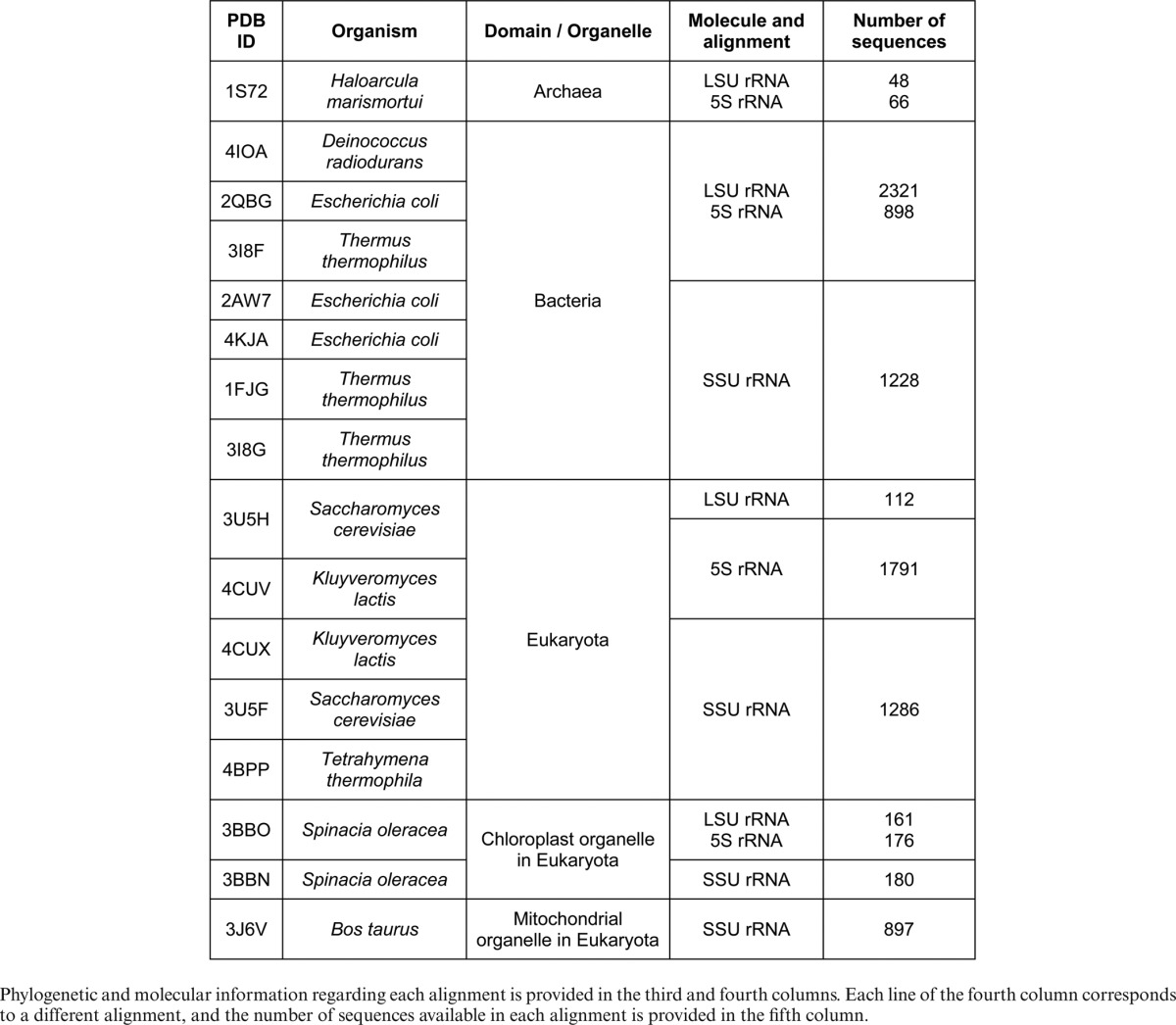
RNA 3D structures currently mapped to alignments and available on R3D-2-MSA, by PDB ID and organism

## RESULTS AND DISCUSSION

### Inputs

#### Manual input

**Figure 3.**
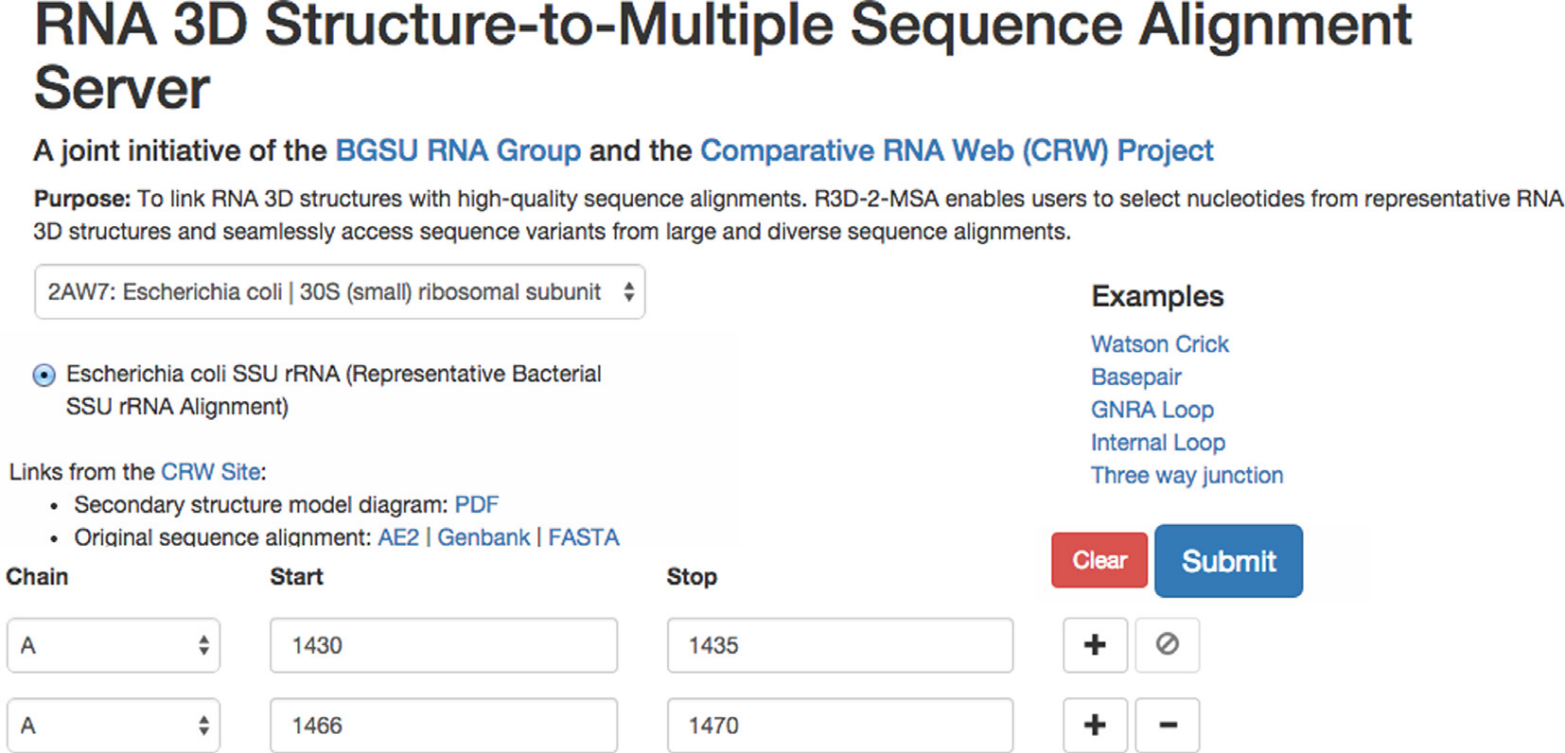
Web page for manual input to the R3D-2-MSA server to select nucleotide ranges corresponding to an internal loop from *E. coli* SSU rRNA (PDB file 2AW7). Upon submission of the request, the server will extract sequence variants from positions in the alignment corresponding to nucleotides 1430–1435 and 1466–1470 in the 3D structure. The input page provides examples for requesting sequence variants of basepairs, hairpin loops, internal loops or multi-helix junctions. Links are provided to display the 2D structure of *E. coli* SSU rRNA or download the complete sequence alignment in any of three formats.

First, the user selects a 3D structure from the drop-down menu, ‘Choose a Structure’. Next, the user chooses which alignment to access, if the structure has more than one associated alignment. For example, LSU generally contain two distinct RNA molecules, the 5S rRNA and the long LSU rRNAs, and so have two associated alignments, of which the user must select one. Since the 5.8S rRNA molecule found in eukaryotes is homologous to the 5’-end of the long LSU rRNA of bacterial, archaeal and organellar ribosomes, it is treated as part of the eukaryal LSU and its sequences are included in the LSU alignment. It is accessed by specifying the appropriate chain ID; other fragmented molecules (for example, chloroplast LSU rRNAs) are treated in the same manner. Where only one alignment is available, as is generally the case for SSU rRNA, it is selected automatically, as shown in Figure 3.

Next, the user specifies one or more ranges of nucleotides in the 3D structure. Each nucleotide range is inclusive and is specified by a chain ID and the starting and ending nucleotides; a range can consist of a single nucleotide or up to 50 nucleotides. To specify a basepair, two single-nucleotide ranges must be entered, unless the bases are adjacent in the sequence. The endpoints of each range must be entered in increasing order, but multiple ranges can be listed in any order. Before transmitting the query to the database, the input page validates that these requirements are met and alerts the user when corrections are required. Queries that include one or more endpoints that have no equivalent position in the ‘lookup sequence’ will fail for reasons explained above. This is indicated with the error message ‘No data available in table’. The user can then modify the query to use an appropriate alternative endpoint, based upon their needs.

#### Programmatic and URL input

The manual input page produces a URL that encodes the query, and visiting this URL prompts the server to produce the query. For example, the query shown in Figure 3 is encoded by the following URL:

http://rna.bgsu.edu/r3d-2-msa?units=2AW7|1|A||1430:2AW7|1|A||1435,2AW7|1|A||1466:2AW7|1|A||1470

One can copy and save this URL to re-generate the output whenever desired. One can also create new query URLs by following this pattern and substituting different nucleotide ranges. The ranges are specified using nucleotide Unit IDs separated by colon and comma delimiters. The first Unit ID in the example above is ‘2AW7|1|A||1430’, which refers to nucleotide 1430, belonging to Chain A of Model 1 in PDB entry 2AW7. The colon between two Unit IDs indicates an inclusive range of nucleotides; a single Unit ID with no colon specifies a single-nucleotide range. Commas are used to separate distinct nucleotide ranges. For use with R3D-2-MSA, the nucleotide identity field (the fourth component of the Unit ID) may be omitted since the server does not use that information; in the case above, R3D-2-MSA would treat ‘2AW7|1|A||1430’ and ‘2AW7|1|A|A|1430’ as identical.

Unit IDs were developed in collaboration with NDB to provide a simple way to uniquely refer to any nucleotide or amino acid in any given macromolecular 3D structure file, whether in the traditional PDB format or in the mmCIF format (2). A full description of the Unit ID format can be found here: http://rna.bgsu.edu/main/rna-3d-hub-help/unit-ids/. In addition to fields for file identifier, model number, chain, residue and residue number, the format supports insertion codes and symmetry operators that are present in some 3D structures. For example, some 3D structures are numbered identically to homologous structures at aligned positions to facilitate structural comparisons. To accommodate insertions in such numbering schemes, insertion codes are used. These are symbols (usually letters) added to the nucleotide number at the point of insertion to provide unique identifiers. For example, the *Thermus thermophilus* SSU rRNA structure 1FJG is numbered to match that of *Escherichia coli;* consequently the nucleotides in the extension of helix 9 are designated sequentially 190A through 190L. To illustrate, the unit ID for nucleotide 190L, a uridine in model 1 of chain A, is 1FJG|1|A|U|190|||L.

#### Programmatic input

Programmatic access to R3D-2-MSA is provided using an API. There are two input modes. First, the API may be used to automatically obtain information regarding which molecules and which of their structures provide access to each of the sequence alignments; this is essentially the data in Table 1 of this paper. Second, the API may be used to query up to five nucleotide ranges from a given 3D structure in a particular alignment. Unit IDs are used to form these ranges, as described above. Use of the API is extensively documented in the help pages, cf. http://rna.bgsu.edu/main/alignment-server-help.

### Outputs

#### Output for human readers

**Figure 4.**
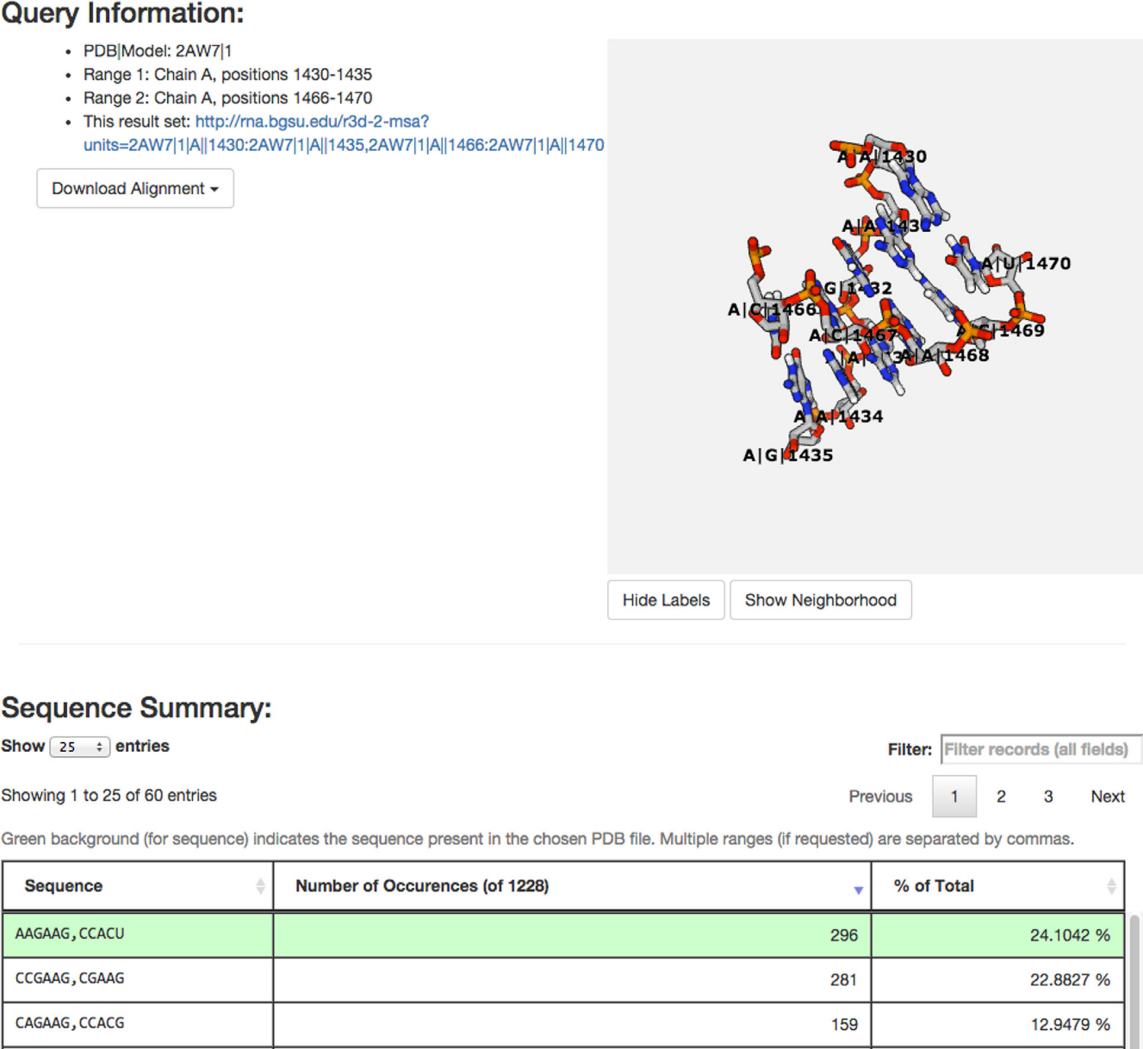
The first two sections of the HTML output page, showing the Query Information component, including the alignment download option and the 3D viewer, and the Sequence Summary component. The first sequence in the Sequence Summary is highlighted in green because it is identical to the sequence in the reference 3D structure.

**Figure 5.**
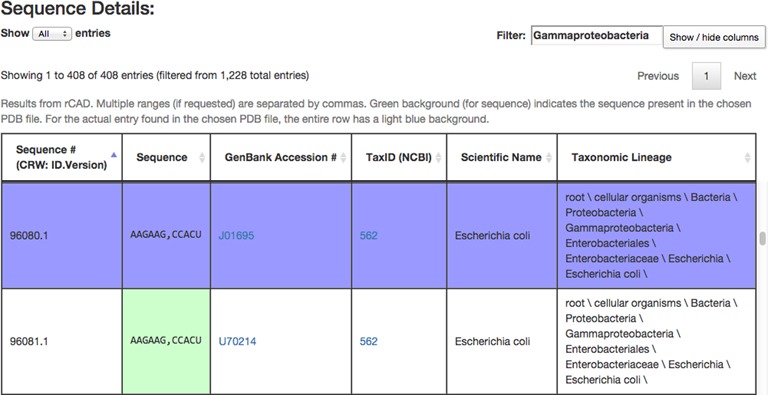
The Sequence Details component of the HTML output page, showing two separate rows of the output. The filter box was used to restrict the display to class Gammaproteobacteria. The row highlighted in blue corresponds to the sequence in the alignment that is closest (in this case, identical) to the sequence from the 3D structure file 2AW7. The box highlighted in green has the same sequence as the 3D structure.

Human-readable HTML output can be obtained from the server either by entering the desired nucleotide ranges manually on the input page or by providing the URL that encodes the search. The output web page contains three major components: (i) the ‘Query Information’ component, a summary of the query, (ii) a ‘Sequence Summary’ table listing the distinct sequence variants found in the alignment and (iii) a ‘Sequence Details’ table providing information for each sequence returned and its biological source. Figures 4 and 5 present a portion of the results from the sample query in Figure 3.

The Query Information section (Figure 4) describes the requested data and provides the URL that can be used to re-generate the results. We also provide an interactive (i.e. rotatable, zoomable) view of the queried 3D region in the results page (20). The ‘Download Alignment’ pop-up menu allows users to download their alignments. Currently, alignment data can be downloaded in either clustal or fasta format. JSON and tab-separated value formats are provided to allow users to download all data provided in the Sequence Details table (see below).

Each row of the Sequence Summary table (Figure 4) lists a distinct sequence variant, the number of occurrences of that variant in the alignment and the percentage of the total number of sequences represented by that variant. When present, multiple nucleotide ranges are separated by commas. Clicking on the header of any column sorts this table by the data in that column. Table rows can be filtered by typing in the ‘Filter’ text box, located above the table; for example, typing ‘ACGU’ will show only sequences that contain ACGU (with no gaps). The sequence highlighted in green is the sequence from the input PDB file.

The Sequence Details table, shown in Figure 5, provides a separate row for each sequence in the alignment. This table provides six columns which display (i) the CRW sequence ID, (ii) the sequence extract, (iii) the Genbank Accession number, (iv) the NCBI taxonomy ID, (v) the scientific name of the organism as provided by NCBI and (vi) the taxonomic lineage, also from NCBI. The Genbank Accession number and NCBI taxonomy ID entries are active links to those databases. Sorting and filtering, as described above, are also enabled for the Sequence Details table; Figure 5 shows an example of restricting the display to a desired phylogenetic group. Display of columns can be toggled on and off using the drop-down menu under the ‘Show/hide columns’ button at the top right of the Sequence Details table. By default, only 25 rows are displayed per page of output, and the data can be browsed by page. Alternatively, the number of rows displayed can be increased manually with the ‘Show entries’ pop-up menu at the top and bottom left of the table. As with the Sequence Summary, sequences highlighted in green match the sequence from the input PDB file. Additionally, the ‘lookup sequence’ used to map the PDB sequence onto the alignment is highlighted in blue.

#### Programmatic output

In response to programmatic input, R3D-2-MSA provides output in JSON format, as described in detail in the help pages, cf. http://rna.bgsu.edu/main/alignment-server-help. This output includes the same information displayed on the HTML page, but without pagination. The JSON output is identical to that obtained by selecting the JSON option from the ‘Download Alignment’ pop-up in the ‘Query Information’ section of the web-based output.

### Updates, extensions and improvements

Over time, the 3D structure database will continue to grow and new structures will be added to the NR lists maintained by BGSU and NDB. Accordingly, the scope of the R3D-2-MSA server will continue to grow. For example, during review and revision of this manuscript, the complete, atomic-resolution human and porcine mitochondrial ribosome structures became available (21,22). We also plan to continue improvements to the server's error handling routines that will enhance its functionality for its users.

Secondary structure (2°) diagrams are a universally accessible way of visualizing large structured RNAs. We provide links to currently available 2° structure diagrams and will produce more as new 3D structures are solved. A natural extension of the alignment server interface is to allow users to make queries based on the sequence positions in 2° structure diagrams for which no 3D structure is currently available, for example, archaeal SSU rRNA.

The Gutell group continues to add sequences to existing alignments, as new genomes are sequenced and new phylogenetic groups are identified. New sequences are selected on the basis of the criteria listed above. In addition, we anticipate expanding the coverage of R3D-2-MSA to 3D structures and alignments of other structured RNA molecules, including Group I and II self-splicing introns, RNase P, riboswitches of various types, signal recognition particle RNA and other structured RNAs. Readers who would like to contribute high-quality, curated alignments that meet the criteria established for the R3D-2-MSA server should contact the authors.

The R3D-2-MSA server was inspired by the RNA 3D coordinate server hosted at BGSU, which uses a similar programmatic access methodology to serve up 3D coordinates of fragments of 3D structure files for local display. R3D-2-MSA calls on the coordinate server to retrieve 3D coordinates, which are then displayed with Protein Viewer. For more details about the coordinate server, the reader is referred to https://github.com/AntonPetrov/jmoltools.

## CONCLUSION

The R3D-2-MSA server provides seamless, nucleotide-level access to high-quality, curated sequence alignments for rRNAs of all major phylogenetic domains, using residue numbers taken directly from representative, atomic-resolution 3D structures selected from PDB/NDB for completeness and quality. R3D-2-MSA is extensible to additional classes of structured RNAs. Its programmatic interface facilitates the use of RNA sequence variability data in a wide range of bioinformatic applications, including RNA 3D modeling, evolutionary studies and identification of non-coding RNA genes in genomes. The server also provides an attractive, easy-to-use interface for manual use by bench scientists and students learning about RNA structure and evolution.
